# A Difficult-To-Diagnose Case of American Tegumentary Leishmaniasis

**DOI:** 10.7759/cureus.44971

**Published:** 2023-09-10

**Authors:** Fady Khoury, Jaime E Campos

**Affiliations:** 1 Medical School, American University of Antigua, St. John's, ATG; 2 Clinical Sciences, Florida International University, Miami, USA; 3 Public Health, Florida International University, Miami, USA; 4 Infectious Disease, Hialeah Hospital, Hialeah, USA

**Keywords:** skin ulcer, cutaneous leishmaniasis, skin eruptions, skin infections, visceral leishmania, medical and diagnostic microbiology, infectious disease diagnosis, infectious disease medicine, american tegumentary leishmaniasis, leishmania guyanensis

## Abstract

This case report presents a difficult-to-diagnose case of American tegumentary leishmaniasis (ATL) caused by *Leishmania (Viannia) guyanensis* in a 24-year-old Hispanic male with a travel history to the Panama jungle, an endemic region for tropical infectious diseases. The patient initially presented with persistent skin lesions that progressed to abscesses with ulceration. Despite negative initial diagnostic tests, including microbiological investigations and histopathological examination, a comprehensive diagnostic workup and subsequent polymerase chain reaction (PCR) confirmed the presence of Leishmania parasites. This case underscores the need to consider tropical infectious diseases despite initial negative tests. Accurate species identification is vital for proper drug treatment, with miltefosine as an emerging option. Early, precise diagnosis and tailored management are essential for successful treatment. This report emphasizes the significance of conducting a comprehensive diagnostic workup, including PCR, in individuals with a history of travel to endemic regions, to accurately diagnose and effectively manage complex infectious diseases.

## Introduction

Leishmaniasis is a parasitic disease caused by diverse species of Leishmania and transmitted through female phlebotomine sandflies [1]. Historically prevalent in Central America, South America, the Indian subcontinent, and Africa, this affliction has long been regarded as a neglected tropical disease [1]. Nonetheless, the global landscape of leishmaniasis is undergoing swift transformations, with emerging cases now being documented in unexpected regions such as the United States, likely influenced by factors like immigration patterns and the evolving climate [2].

Leishmaniasis encompasses two distinct clinical variants, namely visceral leishmaniasis (VL) and cutaneous leishmaniasis (CL) [3]. The pathogenesis of this disease is intricately tied to the delicate balance between TH1 and TH2 CD4+ helper cells. Being an intracellular parasite, the protozoan's fate is determined by the prevalence of a predominantly TH1 response, resulting in effective cell lysis and subsequent elimination of the intracellular parasite. However, this cellular destruction often manifests as a cutaneous presentation and tends to produce milder or even self-limiting symptoms in some cases. On the other hand, individuals exhibiting a TH2-biased response are prone to a more severe visceral presentation due to inadequate intracellular response mediated by antibodies [4].

VL represents a grave clinical condition characterized by a systemic infection involving the liver, spleen, hematologic, and immune systems. Affected patients typically present with subjective symptoms such as recurrent fevers, abdominal pain, unintentional weight loss, and fatigue [3]. In severe cases, cachexia, hepatosplenomegaly, pancytopenia, and hypergammaglobulinemia may manifest [5]. Additionally, individuals with HIV infection are susceptible to opportunistic Leishmania infections owing to the intracellular nature of the protozoan [6]. Notably, post-kala azar dermal leishmaniasis may arise a few months after recovering from acute illness, presenting as facial papules or nodules. Hypopigmentation is also occasionally observed [7].

CL primarily affects the skin and mucosal surfaces, exhibiting a broad spectrum of presentations ranging from isolated mucosal involvement (ML) to localized cutaneous and diffuse cutaneous forms [8]. The cutaneous variant usually begins as a papule at the site of a sandfly bite, gradually enlarging and ulcerating over several weeks to months. It often exhibits raised borders and the potential for abscess formation. Spontaneous resolution of these lesions can occur over a few months, while some studies suggest the possibility of cutaneous lesions metastasizing to mucosal areas. Notably, the mucosal form is often considered the most destructive, capable of causing cartilage and even bone destruction. In individuals with HIV, diffuse cutaneous or mucosal lesions are more commonly observed [9]. Furthermore, a distinct presentation known as leishmania recidivans involves satellite lesions around previously healed CL scars.

Accurate species identification is crucial for the effective management of leishmaniasis, as various species have demonstrated resistance to first and second-line drugs. Standard diagnostic methods encompass microscopy, culture, biopsy, and polymerase chain reaction (PCR). The sensitivity and specificity levels of PCR tests are 97.78% and 61.82%, respectively [10]. Indirect techniques such as the latex agglutination test and rK39 rapid diagnostic tests have been developed to detect specific antibodies, although their utility is primarily focused on diagnosing VL [3].

Historically, antimonials have served as the first-line therapy for leishmaniasis. However, the emergence of drug resistance and associated adverse effects has led to the development of newer agents, including miltefosine, to address these challenges.

This case report details the clinical presentation of a 24-year-old Hispanic patient diagnosed with CL caused by *Leishmania (Viannia) guyanensis*. *L. (V.) guyanensis* infection often presents with clinical features such as persistent skin lesions that progress to abscesses with or without ulceration and raised borders. This case report emphasizes the significance of considering tropical infectious diseases, even in cases where initial diagnostic tests yield negative results. It also underscores the importance of conducting a comprehensive diagnostic workup, particularly in individuals with a history of travel to endemic regions.

## Case presentation

A 24-year-old Hispanic male presented to the emergency department, reporting persistent skin lesions despite receiving outpatient antibiotic treatment. The patient described the presence of multiple lesions on his left leg over the past four weeks. He noted a history of exposure to numerous insect bites, with the lesions initially appearing as red dots accompanied by mild pain and swelling in the leg. Subsequently, the lesions progressed to abscesses with ulceration. These skin lesions initially manifest as papules, which are raised, small, and often painless lumps on the skin. Over time, these papules can progressively enlarge and become larger nodules or plaques. As the disease advances, some of these nodules or papules can undergo ulceration, a process in which the skin tissue breaks down, creating open sores or wounds. These ulcers are typically characterized by a loss of skin integrity, leading to the exposure of underlying tissues. They can vary in size and depth, and they may be accompanied by redness, inflammation, and drainage of fluids. The ulcers associated with *L. (V.) guyanensis* infection often have raised or elevated borders, contributing to their distinctive appearance. The patient denied any significant medical or surgical history, except for recent travel to the Panama jungle.

**Figure 1:**
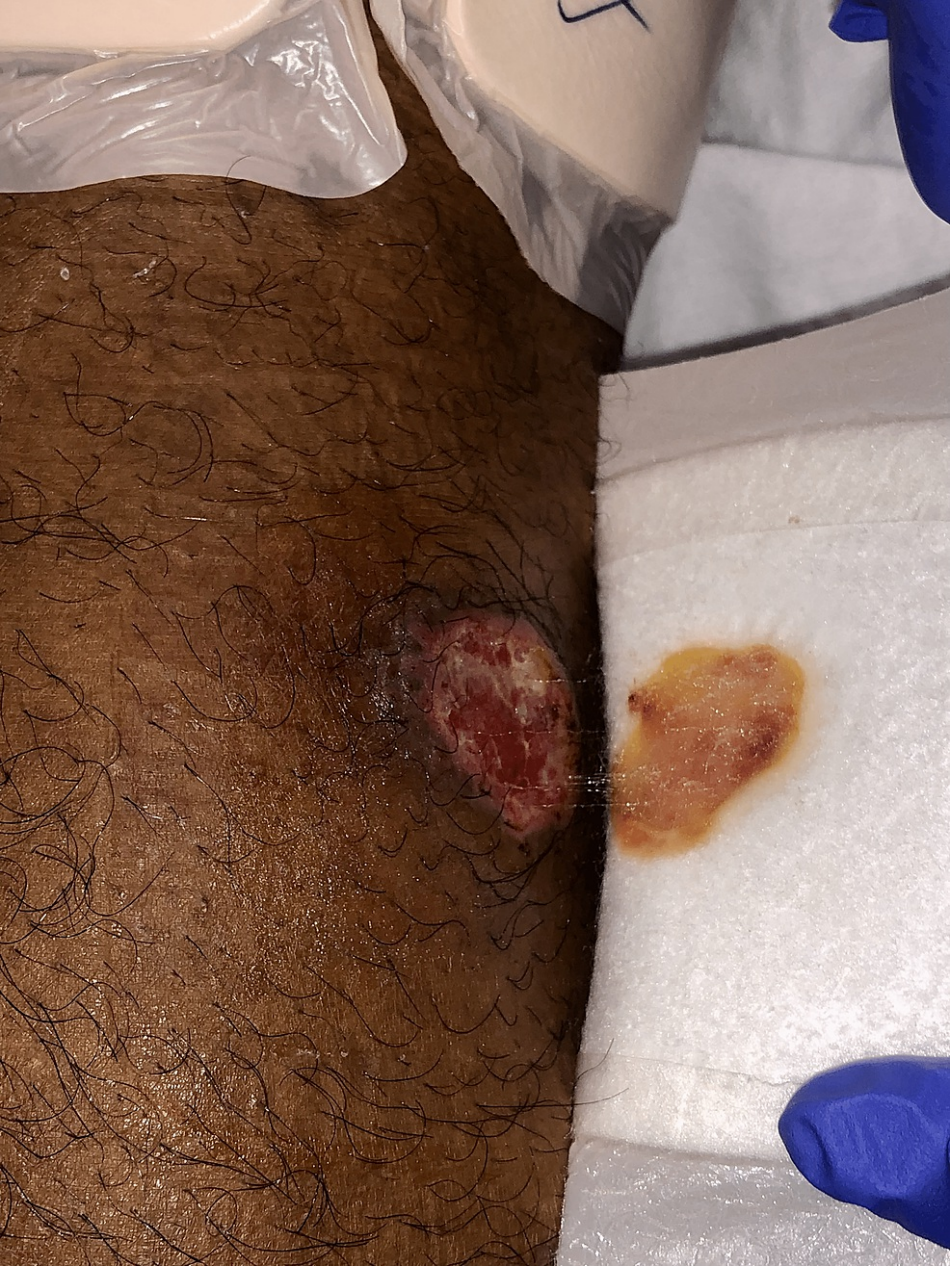
A 3cm abscess with ulceration in the left anterior knee

**Figure 2:**
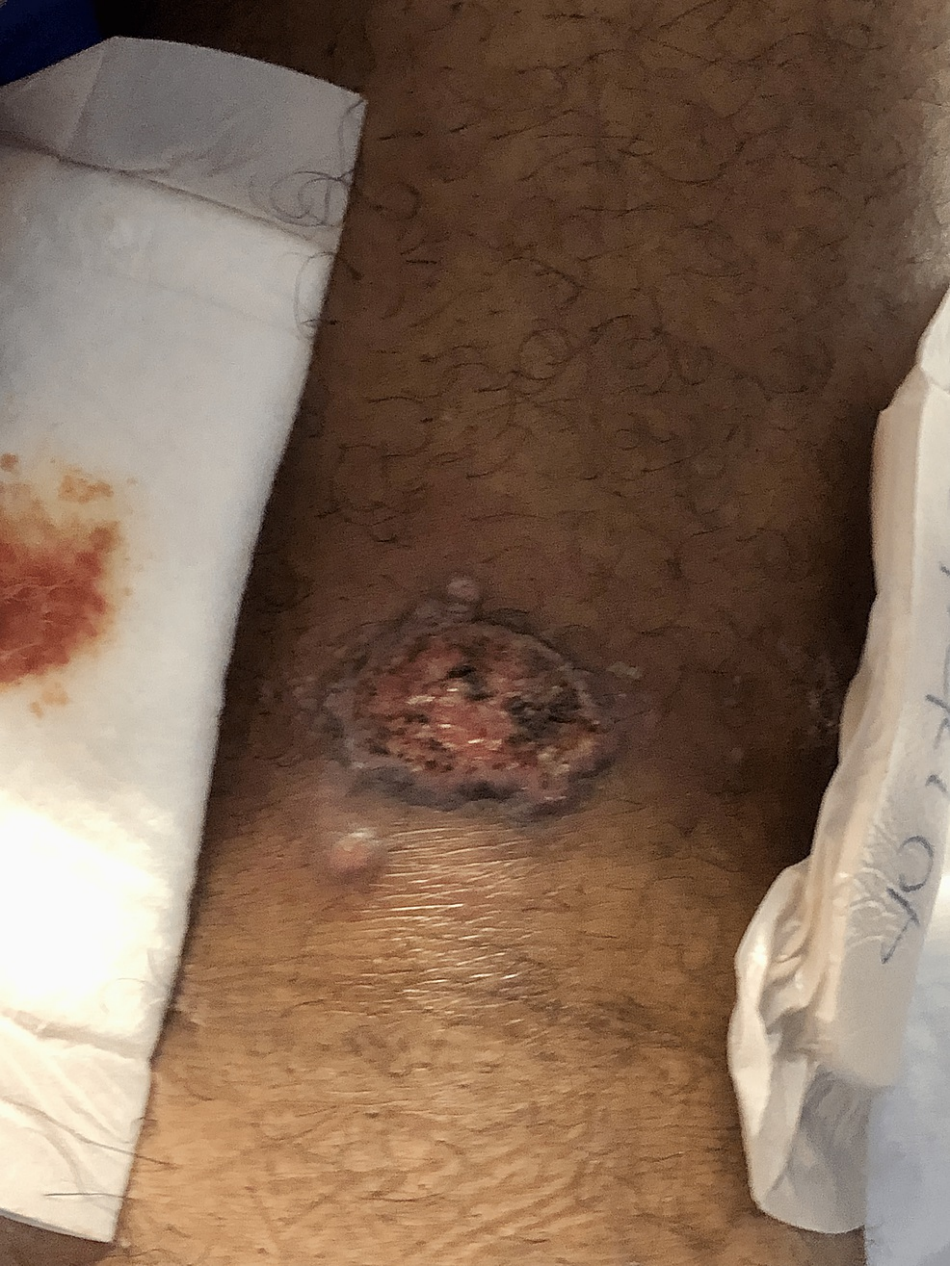
A 3cm abscess in the left posterior thigh

**Figure 3:**
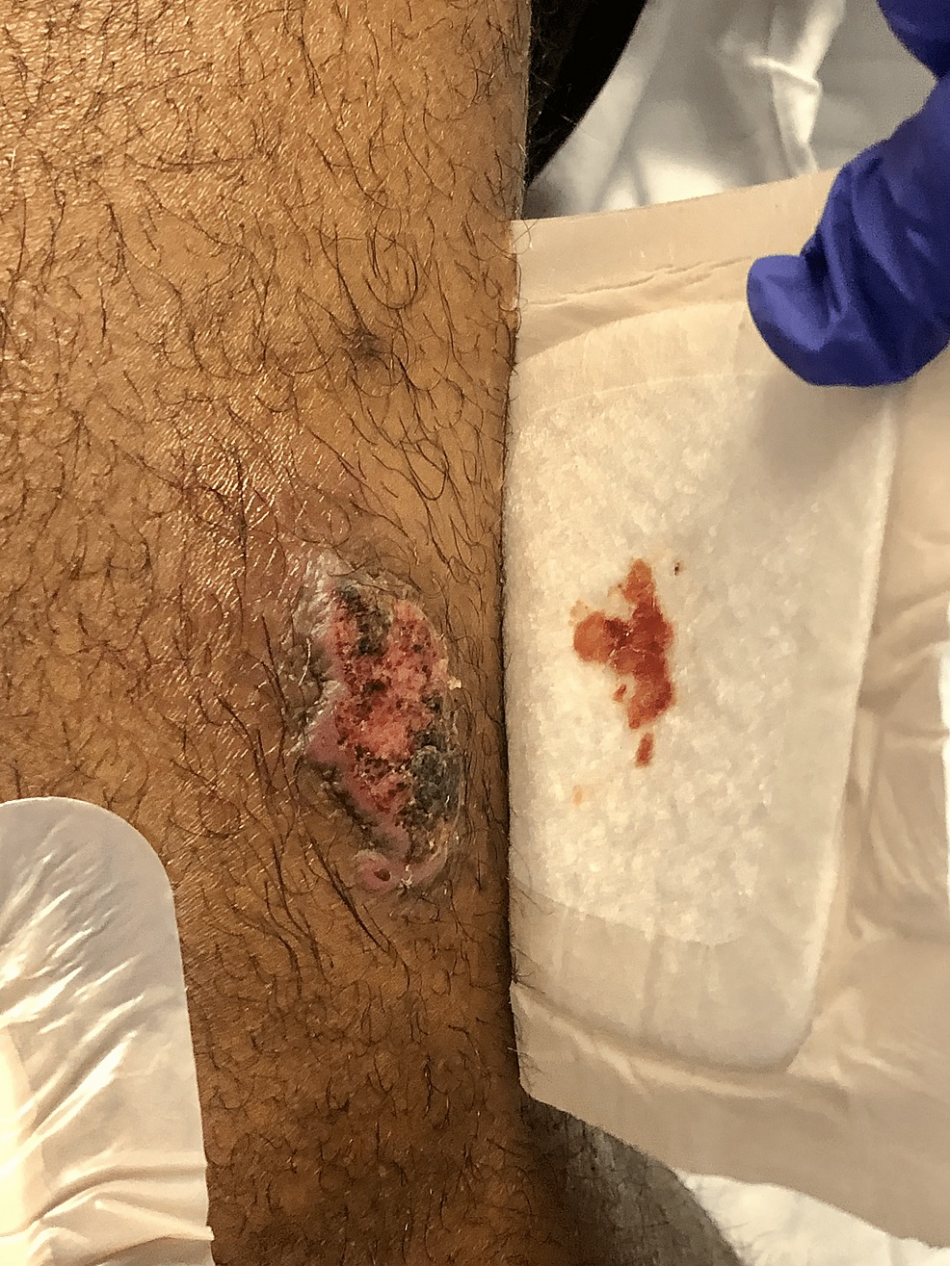
A 3cm abscess in the left lower extremity

**Figure 4:**
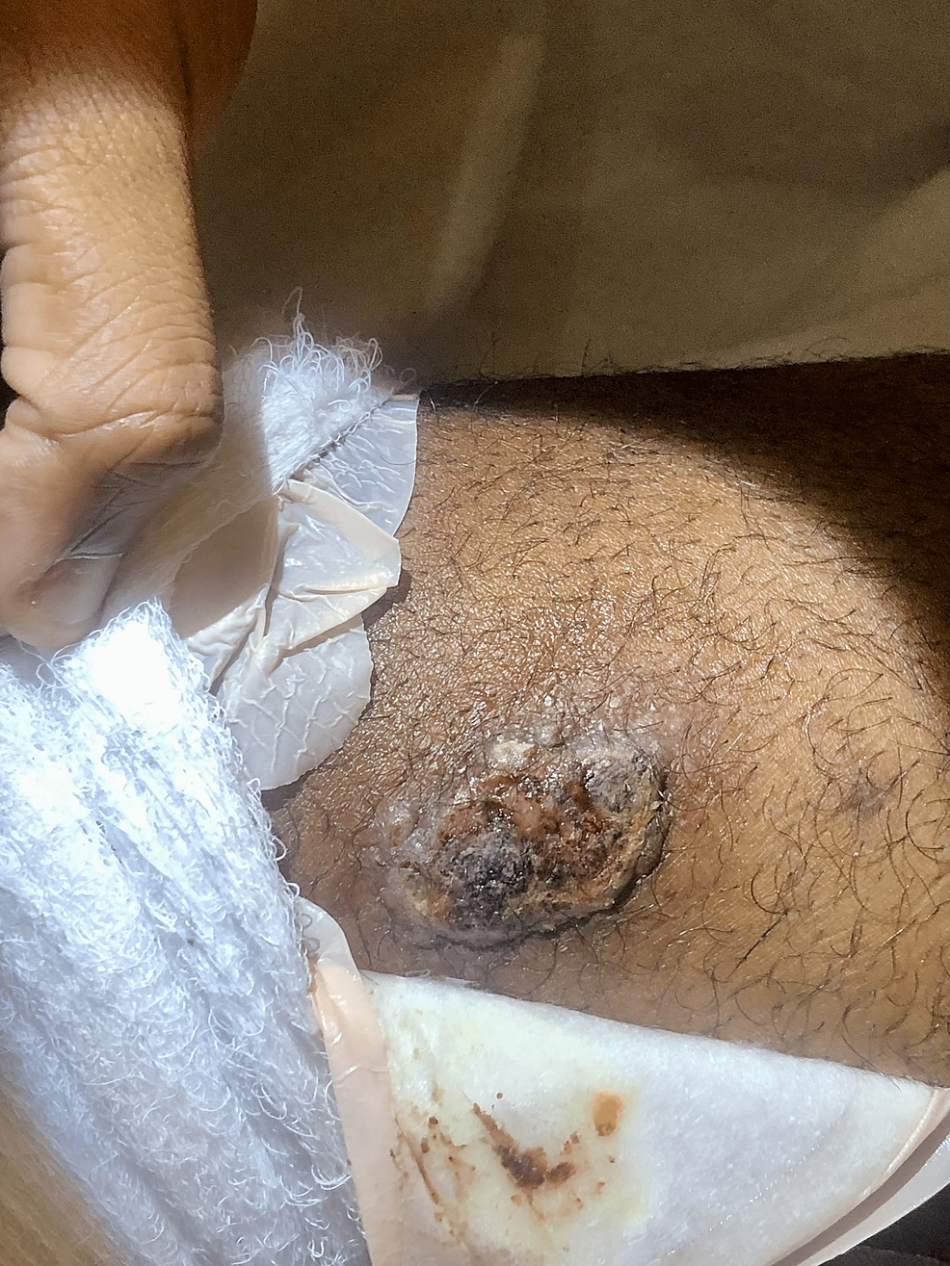
A 3cm abscess on the left anterior distal thigh

Upon physical examination, the patient's vital signs were within normal limits, and his general physical examination did not reveal any notable abnormalities. However, upon dermatological examination, 3cm well-demarcated ulcers with indurated borders were observed on the left anterior knee (Figure 1), left posterior thigh (Figure 2), left lower extremity (Figure 3), and left anterior distal thigh (Figure 4). 

Microbiological investigations, including initial wound culture, blood culture, and anaerobic wound culture, yielded no growth. Acid-fast bacilli (AFB) and gram stains showed no identifiable organisms. The skin biopsy exhibited features of acute and chronic inflammation, characterized by poorly formed necrotizing and non-necrotizing granulomas with giant cells. Special staining using Giemsa failed to detect any Leishmania organisms, and subsequent immunohistochemical stains excluded herpes simplex virus (HSV) 1 & 2 and cytomegalovirus (CMV). Diagnosing Leishmania through skin biopsy using Giemsa stain, it is noteworthy that the sensitivity hovers around 87%, while the specificity approaches nearly 100% [11]. Grocott's methenamine silver (GMS) and periodic acid-Schiff (PAS) special stains revealed the presence of small to large yeasts with narrow-based budding. However, subsequent tests for histoplasmosis, schistosomiasis, and Beta-(1,3)-D-Glucan returned negative results, effectively ruling out atypical bacterial and fungal infections.

Several factors can lead to immune suppression, making the individual more susceptible to leishmaniasis infections. Potential causes include medication adverse effects, malnutrition, and coinfections. Laboratory analyses indicated leukopenia and neutropenia, which were suspected to be consequences of immune suppression resulting from the active infection. Liver function tests (LFTs) and metabolic panels were performed and demonstrated no significant abnormalities. A thorough review of the systems did not reveal any additional complaints. This suggests that the patient did not report or exhibit any other significant health issues or clinical findings beyond the primary concern of Leishmania-induced ulcerations.

The patient's management involved intravenous administration of Vancomycin at a dose of 1000mg/250ml sodium chloride (NaCl) every 8 hours. Initially, fluconazole was initiated; however, it was subsequently discontinued upon the identification of budding yeast. Vancomycin and fluconazole were administered as the initial therapeutic agents preceding the confirmed diagnosis of leishmaniasis. Liposomal amphotericin B was initiated but had to be discontinued due to an immediate adverse reaction. After completing inpatient antimicrobial therapy, the patient was discharged with instructions for home wound care and scheduled follow-up with the infectious disease service at the hospital.

Subsequently, the PCR sample confirmed the presence of *L. guyanensis*, leading to the initiation of outpatient treatment with miltefosine. A significant improvement was observed during the follow-up visit after 4 weeks.

## Discussion

This case report presents a challenging diagnostic case of CL caused by *L. (V.) guyanensis* in a 24-year-old Hispanic male who recently traveled to the Panama jungle, an endemic region for various tropical infectious diseases. The pathogenesis of American tegumentary leishmaniasis (ATL) caused by *L. guyanensis* involves direct parasitic effects, extracellular vesicles, and, in some cases, infection by Leishmaniavirus 1 (LRV1) within the parasite [12].

Leishmania promastigotes are transmitted to the dermis of vertebrate hosts during the blood meal of female phlebotomine vectors. These promastigotes are recognized by Toll-like receptors 4 and 9 (TLRs) and nucleotide-binding oligomerization domain (NOD)-like receptors 1 and 2. Subsequently, phagocytes phagocytize and activate macrophages (MΦ), which then differentiate into antigen-presenting cells (APCs) capable of interacting with the innate and adaptive immune response. Depending on the microenvironment, promastigotes can activate MΦ-M1, leading to the production of nitric oxide (NO) and pro-inflammatory cytokines, or MΦ-M2, resulting in the production of urea, polyamines, and anti-inflammatory cytokines. Processed parasite antigens (Ags) complexed with molecules of the major histocompatibility complex (MHC) are presented on the surface of APCs to activate T lymphocytes. This activation drives the adaptive immune response through recognition by cytotoxic CD8+ and T helper CD4+ lymphocytes via class I (MHCI) and class II (MHCII) molecules, respectively.

Leishmania parasites modulate the host's immune system by selectively inhibiting the release of IL-12 and IFN-γ, thereby evading the activation of cytotoxic CD8+ T cells. Instead, the parasites induce infected MΦ to produce anti-inflammatory cytokines such as IL-4, IL-10, and TGF-β, which inhibit MΦ functions and promote parasite persistence. The delicate balance between host and parasite factors that control the activation or deactivation of MΦ determines the fate of intracellular parasites. In our patient's case, the cutaneous presentation suggests an excess of these anti-inflammatory cytokines [13].

Extracellular vesicles (EVs) have emerged as a recently discovered pathogenic mechanism in CL. Silverman et al. (2008) reported that Leishmania EVs possess the ability to transport virulence factors and manipulate the activity of macrophages (MΦ) to evade parasite elimination. This is accomplished by inhibiting the secretion of pro-inflammatory cytokines. The principal virulence factor identified within these vesicles is the 63 kDa glycoprotein (gp63), a metalloprotease capable of modulating transcription factors, including NF-κB, within targeted MΦ. Given the growing body of evidence surrounding this mechanism, it is plausible that our patient's presentation may have involved this pathway. Further investigations are underway to gain a deeper understanding of this mechanism [14].

The presence of LRV1 has been associated with an increased risk of metastasis [15]. Common sites of metastatic involvement include the nasopharynx and nasal mucosa. While local CL often resolves spontaneously, mucosal involvement typically fails to heal without intervention. This underscores the importance of evaluating mucosal surfaces in patients with CL caused by *L. (V.) guyanensis*. In the case of our patient, there was no evidence of mucosal or metastatic lesions, suggesting that timely detection and treatment may have prevented such complications.

The presence of fungal elements in the biopsy specimens initially led us to consider a fungal etiology. Consequently, antifungal medications were administered. However, these treatments proved ineffective as the patient's lesions persisted without improvement. Moreover, subsequent cultures yielded negative results. Consequently, we determined that the fungal elements observed were likely contaminants of the biopsy specimens, prompting us to explore alternative etiologies.

In light of the patient's immunosuppression, we considered the possibility of underlying comorbid conditions such as HIV or hepatitis, which are commonly associated with VL. However, there were no additional clinical features suggestive of VL, and diagnostic tests for other potential causes of immunosuppression yielded negative results. Thus, we concluded that the ongoing infection itself was the likely cause of the observed immunosuppressive state.

The presence of necrotizing granulomas in the skin lesions was an unexpected finding, prompting us to explore the possibility of leishmaniasis as well as other potential pathologies. Following the Infectious Diseases Society of America (IDSA) guidelines, we performed a PCR test, which yielded a positive result, confirming the diagnosis. We concur with the IDSA's recommendation and emphasize the importance of physicians ordering a comprehensive diagnostic workup, including PCR when warranted, to accurately diagnose and manage complex infectious diseases, especially in patients with a travel history to endemic regions.

Accurate identification of the Leishmania species is crucial to initiate appropriate drug treatment based on susceptibility profiles. Miltefosine has emerged as a superior alternative to antimonials for the resolution of skin lesions [16]. However, the presence of typical fungal microorganisms in the skin lesions can be misleading and divert attention from the actual infectious agent, as was the case with leishmaniasis.

## Conclusions

In conclusion, this case underscores the significance of considering leishmaniasis as a potential etiology of skin lesions in individuals with a travel history to endemic regions. The occurrence of fungal contaminants in biopsy specimens can lead to diagnostic confusion, necessitating additional tests, such as PCR, to accurately identify the underlying infection. PCR stands as a highly sensitive diagnostic tool for leishmaniasis, and a comprehensive diagnostic workup that includes PCR may be imperative for the precise diagnosis and effective management of complex infectious diseases. Early and accurate diagnosis, followed by prompt and appropriate management, holds critical importance for achieving successful treatment outcomes. Lastly, distinguishing the Leishmania species is essential to guide tailored drug therapy based on susceptibility profiles, with alternative treatments like miltefosine demonstrating potential superiority over antimonials in the resolution of cutaneous lesions.
